# Sustainable Recovery of Vanadium from Stone Coal via Nitric Acid Oxygen Pressure Leaching

**DOI:** 10.3390/ma18112530

**Published:** 2025-05-27

**Authors:** Keyu Shen, Fei Li, Yuqin Long, Yang Yang, Huan Long, Ruixin Luo, Wenyuan Ma, Jun Hua, Zhaoxia Yang, Ou Zhuo, Feng Gao

**Affiliations:** School of Chemistry and Chemical Engineering, Jishou University, Jishou 416000, China; skyxiyangyufei@163.com (K.S.); lifei__123456789@163.com (F.L.); 19386913054@163.com (Y.L.); 15197414901@163.com (Y.Y.); 19848113583@163.com (H.L.); ll112206251128@163.com (R.L.); 18095481857@163.com (W.M.); huajun615@163.com (J.H.); yangzhaoxiayzx@163.com (Z.Y.); zhuoouhao@126.com (O.Z.)

**Keywords:** recovery of vanadium from stone coal, nitric acid oxygen pressure leaching, nitrogen recycling, response surface optimization

## Abstract

To overcome the low extraction efficiency and environmental concerns associated with traditional vanadium extraction methods, this study proposes an innovative nitric acid oxygen pressure leaching approach integrated with nitrogen recycling. Through systematic single-factor experiments and response surface optimization, key parameters, including nitric acid concentration, leaching temperature, liquid-to-solid ratio, and total pressure, were carefully evaluated and optimized. Under optimal conditions, consisting of 1.5 mol/L nitric acid, a temperature of 127.43 °C, a liquid-to-solid ratio of 5 mL/g, and a total pressure of 2 MPa, the vanadium leaching efficiency reached 73.1%. Cyclic leaching experiments confirmed the feasibility of nitrogen recycling. Characterization analyses by SEM-EDS, XRD, BET, and FTIR revealed that nitric acid oxygen pressure leaching significantly disrupted the mineral lattice structure, altering the coordination environment of metal ions and increasing surface porosity, thereby facilitating efficient vanadium dissolution from stone coal. This study provides valuable insights and establishes a scientific foundation for developing efficient, environmentally friendly, and economically viable vanadium extraction techniques from low-grade stone coal resources, thereby contributing to sustainable mining practices and resource utilization.

## 1. Introduction

Vanadium, valued for its unique chemical properties and versatile oxidation states, is extensively applied in steel manufacturing, vanadium redox flow batteries, chemical catalysis, and aerospace. As a critical strategic resource, it plays a pivotal role in advancing the sustainable development of key industries in China [1,2,3,4].

With the depletion of conventional high-grade vanadium resources, low-grade secondary resources such as stone coal have become essential for vanadium extraction. Vanadium-bearing stone coal, also called vanadium-bearing black shale, is a shale ore with a high vanadium content [5]. China is the world’s richest source of vanadium-bearing stone coal; its vanadium content is generally less than 1%. Given the extensive reserves of stone coal, optimizing vanadium extraction technologies is of considerable significance for enhancing resource utilization and advancing sustainable mining practices [6,7]. Vanadium in stone coal typically exists in lower oxidation states and is incorporated into aluminosilicate minerals through isomorphous substitution, primarily replacing Al^3+^ in muscovite lattices. It may also associate with organic matter. These occurrence forms significantly hinder vanadium extraction [8,9].

In recent decades, several approaches for extracting vanadium from stone coals have been discovered, including calcareous roasting–water leaching and oxidation roasting–leaching [10,11,12]. However, this process is energy-intensive and generates significant amounts of corrosive gases, which pose environmental challenges [13]. In contrast, hydrometallurgical methods for vanadium extraction from stone coal have garnered increasing attention due to their lower energy requirements and reduced environmental impact.

Hydrometallurgical acid leaching typically includes direct acid leaching [14], fluoride-assisted vanadium leaching [15], oxidative acid leaching [16], microbial leaching [17], and oxygen pressure acid leaching [18]. The direct acid leaching method involves dissolving vanadium into the solution through direct contact between the acid and the mineral. This method typically employs high-concentration sulfuric acid for leaching. However, when applied to low-grade ores, it is often limited by low leaching efficiency and high acid consumption, which restrict its effectiveness and scalability in practical applications. To address the limitations of traditional acid leaching, improved methods have been developed by incorporating oxidants and leaching aids (e.g., fluorides, NaClO_3_, MnO_2_), which enhance the oxidative capacity of acids and promote their interaction with minerals, significantly improving metal dissolution and facilitating more efficient vanadium extraction. However, these methods also have drawbacks, including increased operational costs due to the consumption of additives as well as the risk of secondary pollution. Additionally, the accumulation of impurities in the waste liquid complicates subsequent treatment processes.

Oxygen pressure leaching could accelerate the acid leaching process by introducing oxygen under elevated pressure conditions [19], which significantly enhances the mineral dissociation efficiency, making it particularly well-suited for the extraction of vanadium from low-grade stone coal. This study proposes a nitric acid oxygen pressure leaching method that leverages the synergistic effect between nitric acid and oxygen pressure, significantly enhancing oxidative capacity. In comparison to conventional acid leaching techniques, nitric acid oxygen pressure leaching achieves superior vanadium leaching efficiency under milder conditions, including lower temperatures and pressures with lower reagent consumption.

Although nitric acid oxygen pressure leaching significantly improves vanadium extraction under relatively mild conditions, traditional processes inevitably generate nitrate-rich wastewater, posing environmental challenges and necessitating complex denitrification treatments. Notably, the nitrogen oxide (NO_2_) generated during nitric acid leaching can react with oxygen and water to regenerate nitric acid, offering a practical pathway for nitrogen recycling. Based on this principle, this study proposes an innovative “nitrogen recycling” method by introducing oxygen directly into the leaching system, enabling the continuous conversion of nitrogen oxides back into nitrate ions and effectively reducing nitric acid consumption. The approach proposed in this study eliminates the need for high-temperature roasting and enables recycling of the post-leaching solution, thereby minimizing nitrate discharge and simplifying downstream wastewater treatment. This innovative nitrogen recycling strategy improves resource utilization efficiency and presents a cleaner, more sustainable vanadium recovery route compared to conventional approaches.

## 2. Materials and Methods

### 2.1. Materials

Stone coal used in this study was obtained from Gansu Province, China. The chemical multi-element analysis of stone coal is shown in Table 1.

**Figure 1:**
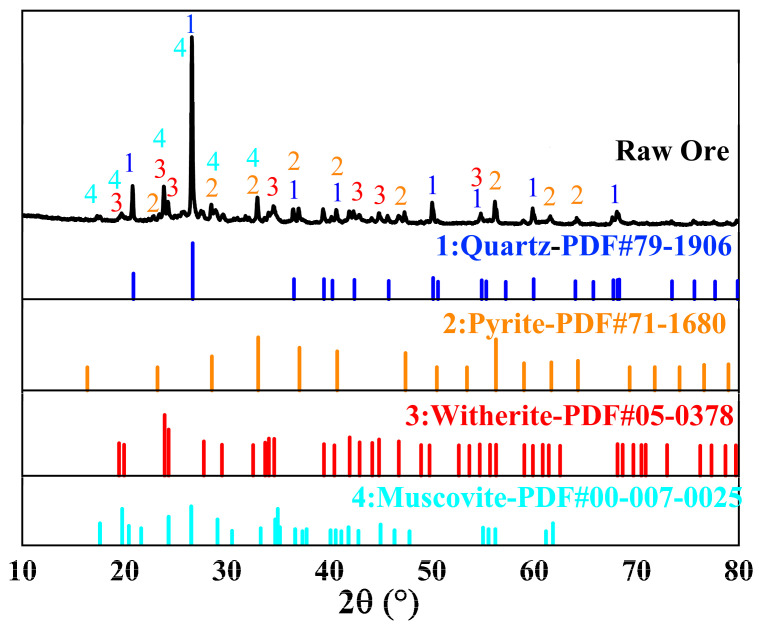
XRD analysis of stone coal.

Figure 1 shows the X-ray diffraction (XRD) pattern of stone coal. The XRD pattern of the raw ore shows that the main mineral components are quartz (SiO_2_), pyrite (FeS_2_), witherite (BaCO_3_), and muscovite (KAl_2_Si_3_AlO_10_(OH)_2_). Quartz is the dominant component, as evidenced by its intense diffraction peaks, reflecting its high stability. Pyrite and witherite are associated with vanadium extraction, potentially releasing vanadium through oxidation and dissolution. While present in lower quantities, muscovite may affect the migration of other elements during leaching.

### 2.2. Experiments and Analytical Methods

**Figure 2:**
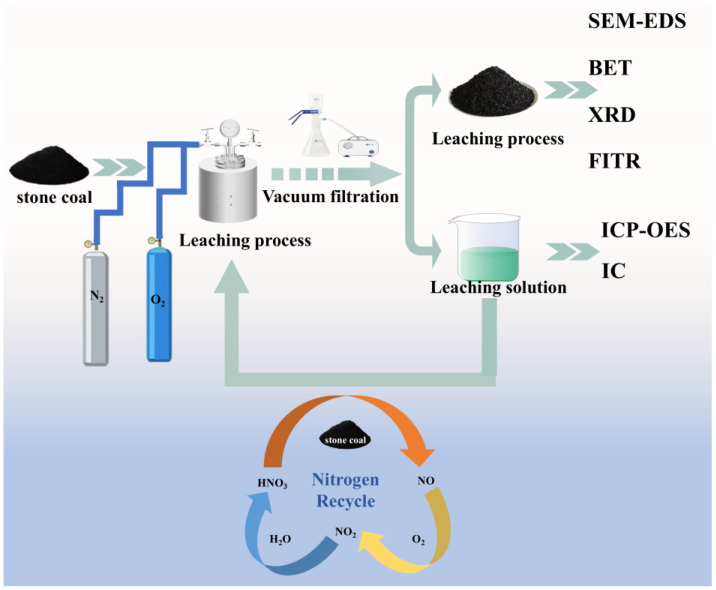
Schematic diagram of the high-pressure leaching process.

The leaching process was conducted in a high-pressure reactor (MSG300-P3-T3-MN1-SV, Hefei Chem Instruments Co., Ltd., Hefei, China). A fixed volume of 100 mL leaching solution with specific acidity was used, and the mass of stone coal was calculated based on the desired liquid-to-solid (L/S) ratio. The temperature was increased at a rate of 5 °C/min to the target value and maintained with stirring for a set duration. Nitrogen or oxygen was introduced into the system at a constant total pressure during the reaction. After leaching, the residue was washed, filtered, dried, and weighed. All experiments were conducted in triplicate. A schematic of the high-pressure leaching process is shown in Figure 2. The vanadium grade in the residue was measured, and the leaching rate was calculated.

(1) $$Y=1-\frac{m_{2}\times \omega_{2}}{m_{1}\times \omega_{1}}\times 100\%$$

where *Y* is the leaching rate of V (%), *m*_1_ is the mass of the raw ore (g), *m*_2_ is the mass of the leaching residue (g), *ω*_1_ is the grade of V in the raw ore (%), and *ω*_2_ is the grade of V the leaching residue (%).

The chemical composition of the stone coal, the residues, and leaching solutions were determined by an inductively coupled plasma optical emission spectrometer (ICP-OES, Avio 200, Shelton, CT, USA). The carbon and sulfur contents in the stone coal were measured with a carbon–sulfur analyzer (Wuxi Gaosu HIR944, Wuxi, China). For mineralogical analysis, the mineral composition of stone coal and leaching residues were analyzed using XRD (Rigaku Miniflex 600, Akishima, Tokyo, Japan), with the conditions of 40 kV tube voltage, Cu target, and 2θ of 10°–90°. The raw ore and leaching residues were examined for their morphological and elemental characteristics using scanning electron microscopy with energy-dispersive spectroscopy (SEM-EDS) (ZEISS Sigma 360, Oberkochen, Baden-Württemberg, Germany). Pore parameters were measured by Brunauer–Emmett–Teller (BET) analysis (Quantachrome nova2000e, Boynton Beach, FL, USA) at N_2_ atmosphere, desorption temperature of 300 °C, and desorption time of 6 h. Fourier-transform infrared spectroscopy (FTIR) (Thermo Fisher Scientific Nicolet iS20, Waltham, MA, USA) was used under conditions of a KBr tablet, scanning wave 400–4000 cm^−1^, and resolution of 4 cm^−1^. Thermogravimetric analysis of the raw stone coal ore was conducted under an oxygen atmosphere using a thermal analyzer (Mettler Toledo TGA2, Columbus, OH, USA), with a temperature range of 30–800 °C and a heating rate of 20 °C/min. Thermodynamic analysis of potential reactions in the system was conducted using HSC 6.0 software.

The concentration of free H^+^ in the aqueous solution was measured through acid–base titration with 0.5 mol/L Na_2_CO_3_ solution, using methyl orange as the endpoint indicator. An aqueous solution with a volume of 5.0 mL was poured into 100 mL conical flask, into which 3 drops of methyl orange indicator was added; after thoroughly mixing, the solution was immediately titrated with the 0.5 mol/L Na_2_CO_3_ solution until a distinct color shift from pink to orange indicated the endpoint [20,21].

In this study, nitrate ions in the leach solution were analyzed by ion chromatography (ICS-5000+, Thermo Fisher Scientific, Waltham, MA, USA) equipped with the IonPac AS19 (4 × 250 nm) separation column. The sample was diluted to remain within the instrument’s linear range and then passed through an anion-exchange solid-phase extraction column to remove potential interfering impurities. A potassium hydroxide (KOH) gradient was used as the eluent at a flow rate of 1.0 mL/min, and the column temperature was maintained at 30 °C. A 25 μL aliquot was injected, and signals were recorded by the suppressed conductivity detector. Before analysis, the leach solution was appropriately diluted to ensure that the nitrate concentration remained within the instrument’s linear detection range. To minimize interference, the diluted sample was pretreated using an anion-exchange solid-phase extraction (SPE) column, which selectively removed potential impurities that could affect nitrate detection.

### 2.3. Optimization of Experimental Design

Response surface methodology (RSM), a type of factorial design, has been widely applied in recent hydrometallurgical research due to its advantages such as statistical robustness, reduced experimental workload, and efficient optimization. Both Central Composite Design (CCD) and Box–Behnken design (BBD) are commonly used to construct second-order (quadratic) models for response variables [22]. Among these, the CCD method is a widely used form of the RSM to optimize the leaching of valuable metals from low-grade resources [23], and CCD is more suitable than BBD for analyzing variable interactions when both the number of factors and levels are high. In this study, RSM based on CCD was employed to effectively design and evaluate the leaching experiments.

The CCD experiment incorporating four factors (nitric acid concentration, leaching temperature, liquid-to-solid ratio, and total pressure) was conducted, and its experimental range was derived from the outcomes of single-factor experiments. A four-factor, three-level experimental design was adopted to optimize the vanadium leaching rate. According to the CCD, a total of 27 runs were conducted. The detailed experimental conditions and results were analyzed using Design Expert 13 software to fit the model through multiple linear regressions. The CCD regression model can be expressed as follows:

(2) $$Y=b_{0}+\sum_{i=1}^{k}\;b_{i}X_{i}+\sum_{i=1}^{k}\;b_{ii}X_{i}^{2}+\sum_{i,j=1,i\neq j}^{k}\;b_{ij}X_{i}X_{j}$$

where Y is the response value (vanadium leaching efficiency); $b_{0}$ is the constant coefficient; $b_{i}$, $b_{ii},$ and $b_{ij}$ are the linear, quadratic, and interaction coefficients, respectively; $X_{i}$ and $X_{j}$ are the coded values of the independent variables; $X_{i}^{2}$ and $X_{ij}$ represent the quadratic and interaction terms, respectively; and *k* is the number of independent variables (in this study, *k* = 4).

## 3. Results and Discussion

### 3.1. Thermal Decomposition Analysis of Stone Coal

**Figure 3:**
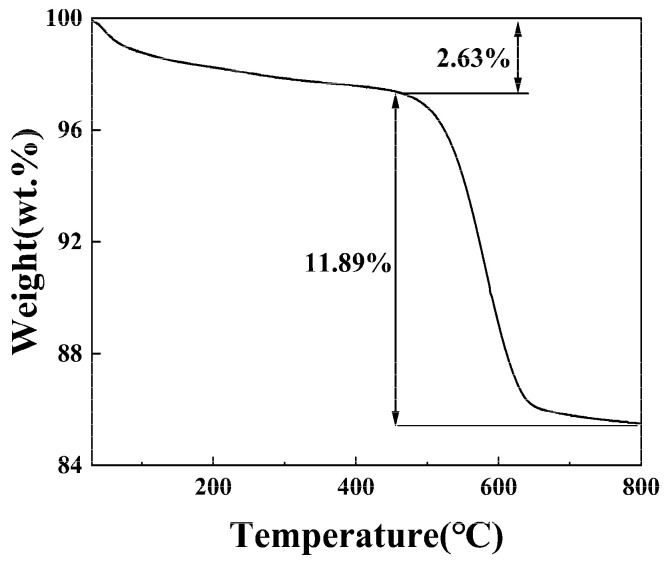
Thermal decomposition analysis of stone coal.

The thermal decomposition of raw stone coal was analyzed to determine weight loss at different temperatures. As shown in Figure 3, the total weight loss from 20 °C to 800 °C is 14.49%. The weight loss process can be divided into two main stages. The first stage occurred between 30 and 450 °C, with a weight loss of 2.63%, primarily due to the evaporation of free and combined water. The second stage occurred between 450 and 800 °C, where the stone coal exhibited a significant weight loss of 11.89%. This was mainly attributed to carbon combustion and pyrite oxidation, which represent the primary reactions in this temperature range.

### 3.2. Comparison of Leaching with Different Methods

A series of comparative experiments were conducted to investigate the effects of different leaching conditions on vanadium extraction from stone coal. All experiments were performed under fixed conditions of t = 5 h, T = 150 °C, L/S = 10 mL/g, and r = 300 rpm. Four conditions were designed to evaluate the influence of gas atmosphere and acid type, as shown in Table 2.

**Figure 4:**
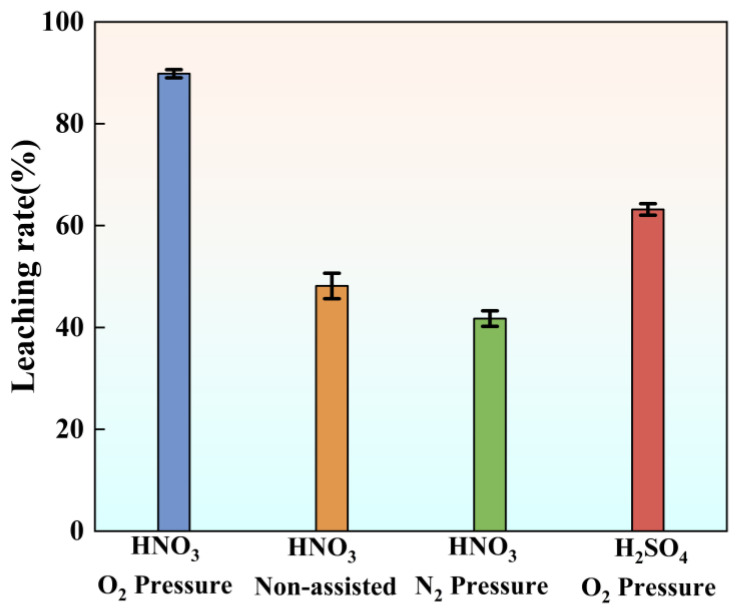
Vanadium leaching efficiency under the different leaching conditions.

Figure 4 illustrates the variation in vanadium leaching efficiencies under different conditions. The highest efficiency of 89.83% was achieved under nitric acid oxygen pressure leaching, as high-pressure oxygen enhances the oxidative power of nitric acid, accelerating the oxidation of vanadium-bearing compounds and improving leaching performance. In comparison, under non-assisted leaching, the leaching efficiency was only 48.14%, a substantial decrease compared to oxygen conditions. This demonstrates the vital role of oxygen partial pressure in enhancing the leaching process. Similarly, under nitric acid nitrogen pressure leaching, the leaching efficiency dropped slightly to 41.75%, indicating that the absence of oxygen significantly limits oxidative reactions, hindering the complete oxidation of vanadium and reducing leaching efficiency. These results suggest that oxygen plays a crucial role in enhancing vanadium leaching efficiency by improving oxidative reaction conditions. Under sulfuric acid oxygen pressure leaching, the leaching efficiency reached 63.19%, surpassing that observed with nitric acid in nitrogen or air but remaining lower than that achieved with nitric acid in oxygen. This comparison highlights that nitric acid exhibits stronger oxidative capacity than sulfuric acid under oxygen pressure leaching conditions, further contributing to the enhanced vanadium leaching efficiency.

Comprehensive analysis indicates that the combination of nitric acid and oxygen enhances both oxidation and acidity, significantly promoting vanadium release from the mineral structure and improving leaching efficiency. Experimental results further demonstrate that gas composition and acid type are key factors influencing the vanadium leaching process.

#### 3.2.1. Phase Transformation Analysis

**Figure 5:**
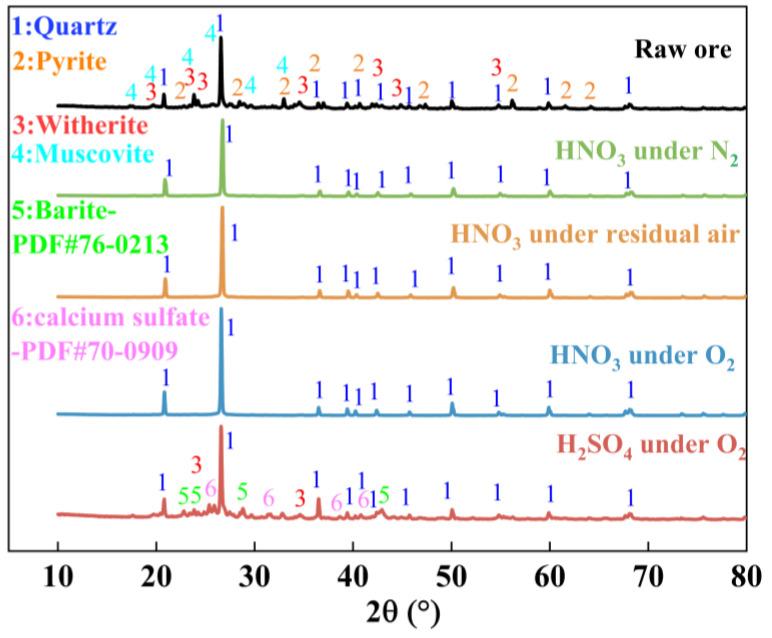
XRD of residues under the different leaching conditions.

XRD analysis was carried out to investigate the phase transformation of stone coal, which revealed the phase transformations and vanadium dissociation mechanisms resulting from the leaching reaction. As shown in Figure 5, compared to the raw ore, the XRD results of the leaching residue under nitric acid oxygen pressure leaching indicate that the diffraction peaks of pyrite in the stone coal disappear completely after the reaction, suggesting its full dissolution under the oxidative action of nitric acid. In contrast, under sulfuric acid oxygen pressure leaching, although pyrite in the stone coal underwent oxidation and its diffraction peaks completely disappeared, the diffraction peaks of barite remained evident in the XRD pattern. This indicates that barium was not entirely dissolved, implying a lower extent of mineral dissolution. The changes in silica were minimal under four leaching conditions, with no significant shifts in the diffraction peaks, indicating its high stability under these conditions. These results suggest that under nitric acid oxygen pressure leaching, mineral dissolution is more thorough.

#### 3.2.2. Micromorphology of Stone Coal Surface

**Figure 6:**
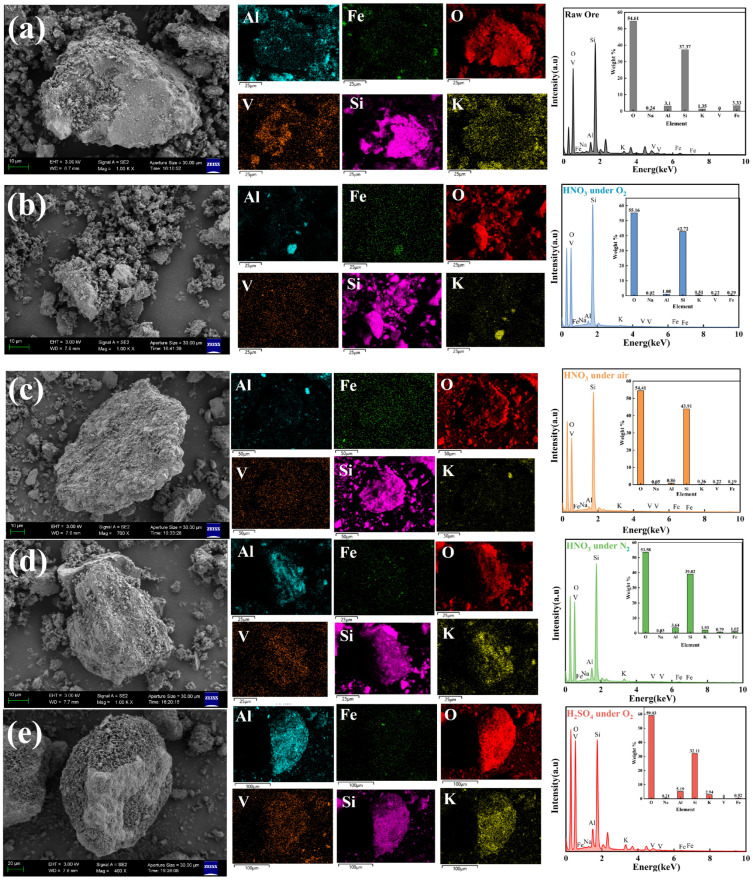
SEM-EDS analysis of stone coal and leaching residues of (**a**) raw ore; (**b**) residue under nitric acid oxygen pressure leaching; (**c**) residue under non-assisted nitric acid leaching; (**d**) residue under nitric acid leaching in nitrogen atmosphere; and (**e**) residue under sulfuric acid oxygen pressure leaching.

SEM-EDS was employed to analyze the microstructural evolution and elemental migration in both the raw ore and leaching residues under four different conditions. Figure 6 illustrates the morphological and elemental changes in the residues, highlighting the influence of leaching conditions on surface dissolution and structure evolution.

Figure 6a shows that the raw stone coal particles have a smooth and compact surface with an intact structure, maintaining an intact structure without any visible pores, suggesting limited mineral dissociation before leaching. Figure 6b shows that after nitric acid oxygen pressure leaching, significant changes occur on the particle surfaces. The particles exhibited significant surface dissolution, reduced particle size, and increased porosity, becoming more irregular and fragmented with numerous cracks appearing. Vanadium distribution was more uniform, with the highest enrichment observed (0.22 wt%). These observations clearly indicate that the synergistic effect between nitric acid and oxygen significantly enhances the oxidative dissolution of stone coal minerals, facilitating vanadium dissociation.

In contrast, Figure 6c shows that residues from non-assisted leaching with nitric acid under residual air conditions display partial dissolution and surface coarsening, with relatively limited vanadium dissociation. Figure 6d shows that, after nitric acid leaching under nitrogen pressure, minimal morphological changes are observed, indicating weaker oxidation due to oxygen deficiency and consequently low vanadium release. Figure 6e shows that, after sulfuric acid oxygen pressure leaching, the particle surfaces exhibit moderate dissolution and slightly enhanced porosity compared to non-assisted and nitrogen-assisted leaching. However, the overall morphological changes remained inferior to those observed under nitric acid oxygen pressure conditions.

These SEM results clearly demonstrate that nitric acid oxygen pressure leaching provides the most favorable conditions for vanadium dissociation. The introduction of oxygen effectively enhances the oxidative dissolution and surface alteration of mineral particles, resulting in enhanced mineral porosity and structural breakdown. Consequently, vanadium dissociation and migration were significantly improved, highlighting the crucial role of oxygen in promoting mineral oxidation and optimizing the overall vanadium leaching efficiency.

#### 3.2.3. Parametric Analysis of Particle Pores

**Figure 7:**
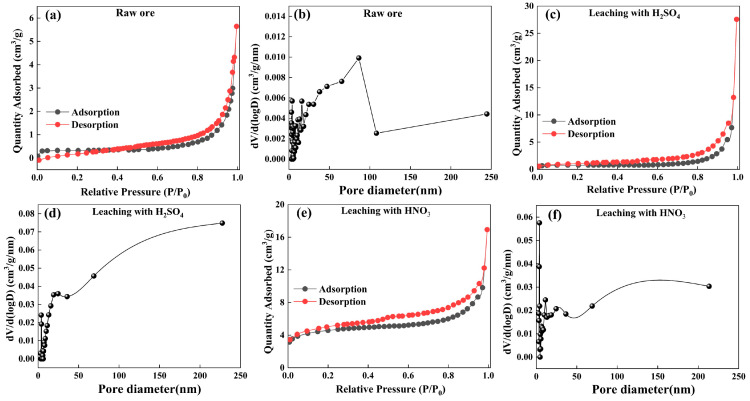
N_2_ adsorption–desorption isotherm and pore size distribution of samples of (**a**,**b**) raw ore; (**c**,**d**) residue under sulfuric acid oxygen pressure leaching; and (**e**,**f**) residue under nitric acid oxygen pressure leaching.

The differences in the pore structures of the stone coal particles before and after leaching are illustrated in the SEM images. To accurately analyze the change rules of these pores, BET analysis was performed. The sample was desorbed at 300 °C in a nitrogen atmosphere for 6 h. The results of the N_2_ adsorption–desorption curve and pore size distribution are shown in Figure 7, and specific surface area, total pore volume, and average pore diameter are shown in Table 3.

The specific surface area of raw stone coal is 0.951 m^2^/g, with a total pore volume of 0.00873 cm^3^/g and an average pore diameter of 3.092 nm, indicating a dense surface structure that limits the full progression of the acid leaching reaction. After the sulfuric acid and nitric acid oxygen pressure leaching treatments, the results are as follows: sulfuric acid oxygen pressure leaching significantly increased the specific surface area to 4.274 m^2^/g, total pore volume to 0.08412 cm^3^/g, and average pore diameter to 3.925 nm, suggesting that sulfuric acid oxygen pressure leaching effectively disrupted the mineral structure, expanded the surface area, and created more pores, thereby improving the contact between the acid and minerals, promoting more contact between the acid and minerals, and enhancing the leaching efficiency. In contrast, nitric acid oxygen pressure leaching showed a more pronounced modification effect, leading to a dramatic increase in specific surface area to 32.297 m^2^/g, total pore volume rising to 0.05804 cm^3^/g, and an average pore diameter of 3.912 nm. This indicates that nitric acid oxygen pressure leaching has a stronger effect on enlarging the mineral surface area and generating more pores, thereby facilitating more efficient vanadium leaching.

The nitric acid oxygen pressure leaching significantly enhanced the specific surface area and porosity of the minerals, exposing more reactive sites and thereby improving the efficiency of vanadium leaching. The total pore volume of the filter residue after nitric acid oxygen pressure leaching was significantly higher than that of the raw ore. While slightly lower than the result from sulfuric acid oxygen pressure leaching, the substantial increase in specific surface area indicates that nitric acid oxygen pressure leaching generated more micropores and mesopores, maintaining a high degree of porosity. This highlights the enhanced ability of nitric acid oxygen pressure leaching to modify the mineral structure, promoting more efficient vanadium dissociation. This result is consistent with the SEM results.

#### 3.2.4. FTIR Analysis

**Figure 8:**
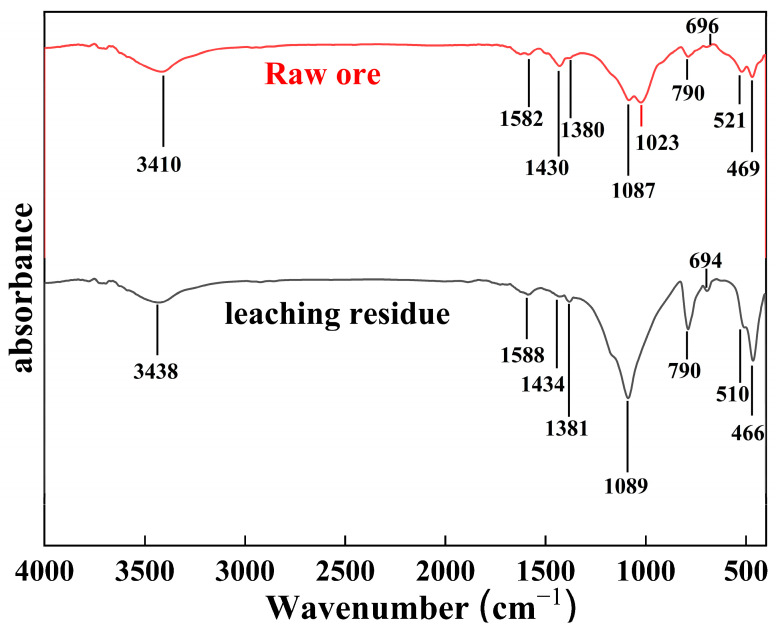
FTIR diagram of raw ore and nitric acid oxygen pressure leaching residue.

To evaluate structural changes in stone coal under nitric acid oxygen pressure leaching, infrared spectra were measured for both raw stone coal and the corresponding leached residues. By comparing the infrared characteristic absorption peaks of the stone coal samples before and after nitric acid leaching, the unique functional groups on the mineral surface were studied, revealing the molecular structure changes in stone coal during the nitric acid oxygen pressure leaching process. Figure 8 presents the relevant IR spectra.

In the spectrum of the raw stone coal, a broad peak at 3410.49 cm^−1^ corresponds to the stretching vibration of -OH groups in water molecules within the mineral structure or the crystal lattice [24], indicating that the stone coal has certain hydrophilic properties, which can be attributed to the presence of hydrophilic muscovite minerals. Additional peaks at 1582 cm^−1^, 1430 cm^−1^, and 1380.78 cm^−1^ primarily reflect the asymmetric, symmetric, and bending vibrations of carboxyl (-COOH) groups, respectively [25]. After nitric acid leaching, these peaks shift to 1588 cm^−1^, 1434 cm^−1^, and 1381 cm^−1^, indicating that while these functional groups remain intact, their polar environment is altered by the oxidative conditions. This observation indicates that nitric acid not only affects the mineral surface and associated organic matter but also modifies their molecular structure rather than simply decomposing them. Additionally, the absorption peak at 1023.89 cm^−1^, primarily associated with witherite (BaCO_3_) [26], was completely absent in the leached residues, confirming the dissolution of carbonate ions during the reaction. These findings demonstrate that leaching under nitric acid oxygen pressure conditions effectively disrupted the crystalline structure of the minerals, leading to pronounced structural transformations.

Furthermore, the strong absorption band at 1086 cm^−1^ in the infrared spectrum of the raw ore is attributed to the antisymmetric stretching vibration of Si(Al)-O(Al) and Si-O-Si(Al) in silica [27,28], while the characteristic peaks at 694 cm^−1^ and 798 cm^−1^ correspond to the symmetric stretching vibration of silicon-oxygen bonds [27,29]. The characteristic peaks at 521 cm^−1^ and 469 cm^−1^ correspond to the bending vibration of Si-O. After leaching [30], these peaks exhibited varying degrees of red or blue shifts, further demonstrating that nitric acid leaching significantly affected the symmetry of the silicate crystal structure.

Due to the stability of silicate minerals in stone coal, vanadium may exist in an isomorphic form by substituting aluminum or silicon in the crystal lattice of aluminosilicates such as muscovite. This structural incorporation makes vanadium difficult to oxidize and dissolve under normal conditions [8]. However, nitric acid oxygen pressure leaching resulted in a distortion of the mineral structure, altering the coordination of internal metal ions. This change reduced the leaching difficulty of metal ions and significantly improved the vanadium leaching efficiency from stone coal.

### 3.3. Nitric Acid Oxygen Pressure Leaching

**Figure 9:**
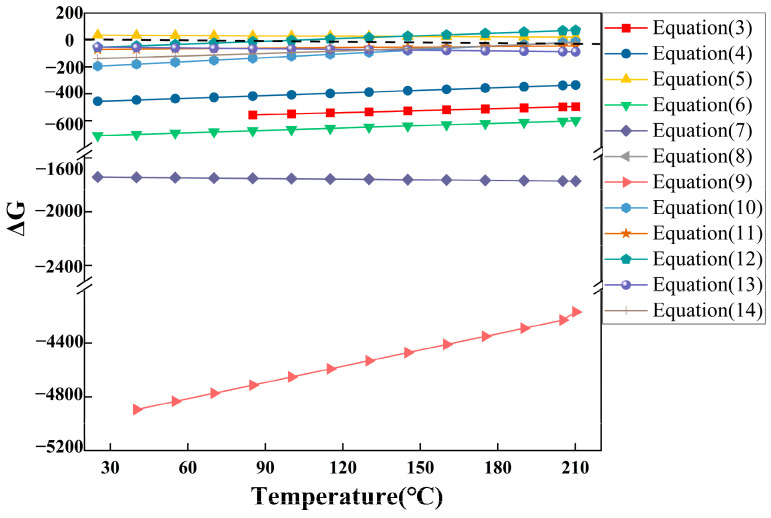
Possible reactions during leaching and the standard Gibbs free energy versus temperature curves.

To identify the critical parameters influencing the nitric acid pressure leaching of stone coal vanadium ore, a single-factor experimental design was employed. The nitric acid concentration, reaction temperature, solid-to-liquid ratio, reaction time, total system pressure, and stirring speed were systematically varied to assess their effects on vanadium leaching efficiency. Thermodynamic calculations of the key reactions were performed using HSC Chemistry 6.0 software. Table 4 and Figure 9 present the primary reactions and the temperature-dependent variation in their Gibbs free energy (ΔG). By integrating reaction equations with thermodynamic analysis, the mechanisms by which these parameters influence leaching behavior are further elucidated. The main oxidation reactions of vanadium appear in Equations (3) and (4) in Table 4. According to the thermodynamic data, the negative ΔG values for these reactions in the 60–180 °C range confirm their spontaneity. Given that the reaction represented by Equation (3) has a lower ΔG than that of Equation (4), it is considered the dominant pathway in the system. The oxidation reactions of sulfur and iron also played a pivotal role in the leaching process. Based on the results from HSC Chemistry 6.0 software calculations, the synergistic effect of nitric acid and oxygen significantly facilitated the oxidation and dissociation of vanadium and iron within the minerals, thereby markedly enhancing leaching efficiency.

#### 3.3.1. Effect of the Nitric Acid Concentration on the Leaching Rate

**Figure 10:**
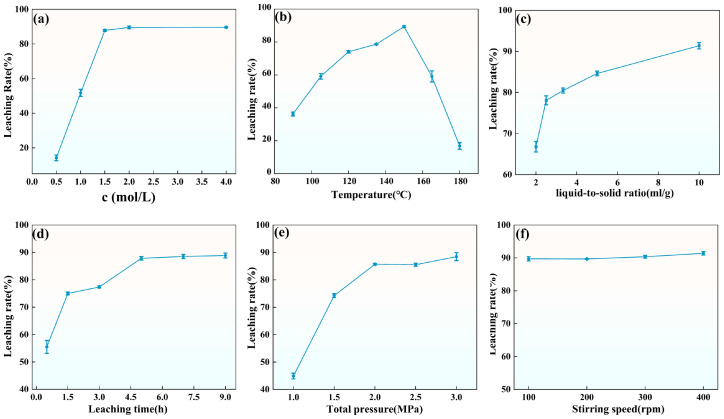
Effects of different acid leaching conditions on the leaching rate of V: (**a**–**f**) The effects of nitric acid concentration, temperature, liquid-to-solid ratio, time, total pressure, and stirring speed on the leaching rate.

The initial nitric acid concentration in the leaching solution significantly influences the vanadium leaching process. As shown in Figure 10a, the experimental results demonstrate a significant increase in vanadium leaching efficiency with increasing nitric acid concentration. At lower concentrations, the relatively weak acidity limits the effective disruption of the mineral structure, resulting in lower leaching efficiency. At a nitric acid concentration of 0.5 mol/L, vanadium leaching efficiency was only 14.52%. As the nitric acid concentration increased, its oxidative capacity was enhanced, significantly promoting vanadium dissociation from the mineral lattice. At 1.5 mol/L, the leaching efficiency reached 87.36%. Further increases to 2.0 mol/L and 4.0 mol/L resulted in leaching efficiencies of 89.70% and 89.35%, respectively, with little additional improvement. Thus, 1.5 mol/L is considered the optimal nitric acid concentration.

#### 3.3.2. Effect of Temperature on the Leaching Rate

Temperature is a critical parameter influencing the oxygen pressure acid leaching reaction, and it is intrinsically linked to the total system pressure. A single-factor experiment was conducted to assess the effect of temperature on leaching efficiency. The experimental results, shown in Figure 10b, demonstrate that vanadium leaching efficiency increases with temperature, with a marked improvement as the temperature rises from 90 °C to 150 °C, reaching a peak of 89.05%. This indicates that higher temperatures facilitate mineral dissolution and vanadium dissociation, thereby significantly enhancing leaching efficiency. However, when the temperature exceeds 150 °C, a decline in leaching efficiency is observed. In a system with a fixed total pressure of 2 MPa, increasing temperature causes a gradual rise in the partial pressures of nitrogen oxides (NO and NO_2_), water vapor, and evaporating nitric acid gases, while the partial pressure of oxygen decreases proportionally. Specifically, for Equation (12) in Figure 9, the ΔG value remains consistently greater than zero, indicating that the reaction cannot proceed spontaneously at elevated temperatures. As a result, the conversion of nitrogen oxides to nitrate ions is inhibited, occupying the gas-phase partial pressure space within the system. This condition results in an insufficient oxygen supply, which limits the progression of oxidation reactions. Consequently, the dissolution and migration of vanadium are impeded, resulting in a decrease in leaching efficiency. This condition results in an inadequate oxygen supply, thereby limiting the progression of oxidation reactions. Consequently, the dissolution and migration of vanadium are substantially hindered, ultimately leading to a reduction in leaching efficiency.

Considering the comprehensive influence of temperature on the leaching reaction, 150 °C is identified as the optimal leaching temperature. At this temperature, the oxygen partial pressure remains stable and sufficiently high, while the reaction rate is moderate, facilitating optimal vanadium leaching efficiency. Moreover, this temperature avoids the detrimental effects associated with higher temperatures, such as a reduction in oxygen partial pressure and a decrease in the spontaneity of the reaction.

#### 3.3.3. Effect of Liquid-to-Solid Ratio on the Leaching Rate

The liquid-to-solid ratio is a critical factor influencing the oxygen pressure acid leaching process. As shown in Figure 10c, as the liquid-to-solid ratio increases, leaching efficiency rises from 67.45% to 90.74%. This observation indicates that a higher liquid-to-solid ratio enhances the contact area between the solvent and the minerals, thereby facilitating a more efficient interaction of nitric acid with the stone coal and promoting vanadium leaching. Based on these results, a liquid-to-solid ratio of 10 mL/g was determined to be the optimal condition.

#### 3.3.4. Effect of Time on the Leaching Rate

Time is a critical parameter in the oxygen pressure acid leaching process. The effect of reaction time on leaching efficiency was evaluated through a single-factor experiment, with the results shown in Figure 10d. At 0.5 h, the leaching efficiency remained relatively low, reflecting incomplete mineral dissolution in the early stage. As the reaction proceeded, efficiency rose from 57.61% to 88.25%, gradually reaching an equilibrium at 5 h. Extending the leaching time beyond 5 h did not significantly improve efficiency, indicating that 5 h represents the optimal duration for this process.

#### 3.3.5. Effect of Total Pressure on the Leaching Rate

This study focuses on nitric acid oxygen pressure leaching of stone coal, where total pressure is maintained by injecting oxygen into the reaction vessel. Therefore, it is necessary to investigate how total pressure affects vanadium leaching efficiency. As illustrated in Figure 10e, increasing the total pressure from 1 MPa to 2 MPa significantly improved leaching efficiency, rising from 67.45% to 83.38%. However, a further increase to 3 MPa provided only a minor improvement, reaching 87.16%.

At lower pressures, the oxygen concentration is insufficient, resulting in a lower oxidation reaction rate and limited dissolution of minerals. Increasing total pressure raises oxygen availability and improves mineral dissolution by enhancing the oxidation process. However, when the total pressure becomes too high, the system approaches oxygen saturation, and additional pressure mainly increases the concentrations of other gases such as nitrogen oxides and water vapor. This prevents further significant increases in oxygen concentration, limiting the benefit of additional pressure. Thus, based on experimental results, a total pressure of 2 MPa is optimal for vanadium leaching.

#### 3.3.6. Effect of Stirring Speed on the Leaching Rate

Stirring speed is a critical parameter affecting the efficiency of the leaching reaction. To investigate its influence on vanadium leaching, single-factor experiments were conducted at stirring speeds ranging from 100 rpm to 400 rpm, and the results are shown in Figure 10f. Throughout this experimental range, the vanadium leaching efficiency remained relatively high. Initially, at the lowest stirring speed (100 rpm), the leaching efficiency was already notably high at 88.86%. As stirring speed gradually increased, efficiency exhibited a steady yet modest rise, reaching 91.38% at 300 rpm. However, when stirring speed exceeded 300 rpm, further increases had negligible effects on efficiency. From a hydrodynamic perspective, under high stirring speeds, convective mixing and turbulence intensity are sufficient to ensure uniform particle dispersion [31]. Under these conditions, additional increases in stirring speed do not significantly enhance mass transfer or leaching efficiency. Therefore, 300 rpm was selected as the optimal stirring speed.

#### 3.3.7. Response Surface Optimization of Acid Leaching Systems

The response surface optimization design of the acid leaching system was conducted. Since the experiments are conducted in a high-pressure reactor, the heating and cooling times required vary with different experimental conditions. If time was included as a variable in the optimization, it could introduce significant interference, potentially compromising the accuracy and reliability of the results. Since the time and stirring speed variables have less influence on the overall system after stabilization, nitric acid concentration, temperature, solid–liquid ratio, and total pressure were selected for optimization to achieve the best acid leaching effect. The ranges of the tested variables were as follows: nitric acid concentration ranged from 0.5 mol/L (low factor value) to 2.5 mol/L and 3.1 mol/L (high factor values), with a center point of 1.5 mol/L; temperature ranged from 70 °C and 90 °C (low factor values) to 150 °C and 170 °C (high factor values), with a center point of 120 °C; solid–liquid ratio ranged from 2 to 5 and 6.5 (high factor values), with a center point of 3.5; and total pressure ranged from 1.5 MPa (low factor value) to 2.5 MPa and 3.0 MPa (high factor values), with a center point of 2 MPa. Specific experimental variables are shown in Table 5. A Central Composite Design (CCD) was used for the experiments, with all experiments conducted in triplicate. Data analysis was performed using Design Expert 13 software. The results are presented in Table 6, and the best-fit models to the response surfaces are shown in Equation (15):

Y = −0.906284 − 0.145933A + 0.0162179B + 0.0592603C + 0.423698D − 0.00002AB + 0.0100083AC  −0.003525AD − 0.000086BC − 0.000735BD − 0.00546667CD
+ 0.0557098A^2^ − 0.000056B^2^  −0.00319392C^2^ −
0.0632953D^2^                          (15)

The response surface analysis of the acid leaching system was carried out through an analysis of variance (ANOVA) on the leaching rate model. As presented in Table 7, the ANOVA results indicate that the factors of models A, B, C, D, AC, BC, BD, CD, A^2^, B^2^, and C^2^ are D^2^ < 0.05 (at the 95% confidence level, *p* < 0.05 was considered statistically significant and *p* < 0.0001 was considered statistically highly significant) [14,20,21], indicating that the effects of the nitric acid concentration, leaching temperature, liquid–solid ratio, total pressure, as well as the interactions between these factors and their squared effects on the vanadium leaching efficiency were significant. The *p*-values for these factors were all less than 0.05, suggesting that these factors play a crucial role in determining the efficiency of the vanadium leaching process. The F-value of the whole model is 396.59, with *p*-value < 0.0001, indicating that the model is statistically significant. Furthermore, the lack-of-fit parameter yielded an F-value of 2.46 and a *p*-value of 0.1697, which is greater than 0.05, demonstrating that the lack-of-fit parameter was statistically insignificant. The signal-to-noise ratio of the model is 90.9203, which is well above the threshold of four, suggesting high credibility of the model and reasonable data.

**Figure 11:**
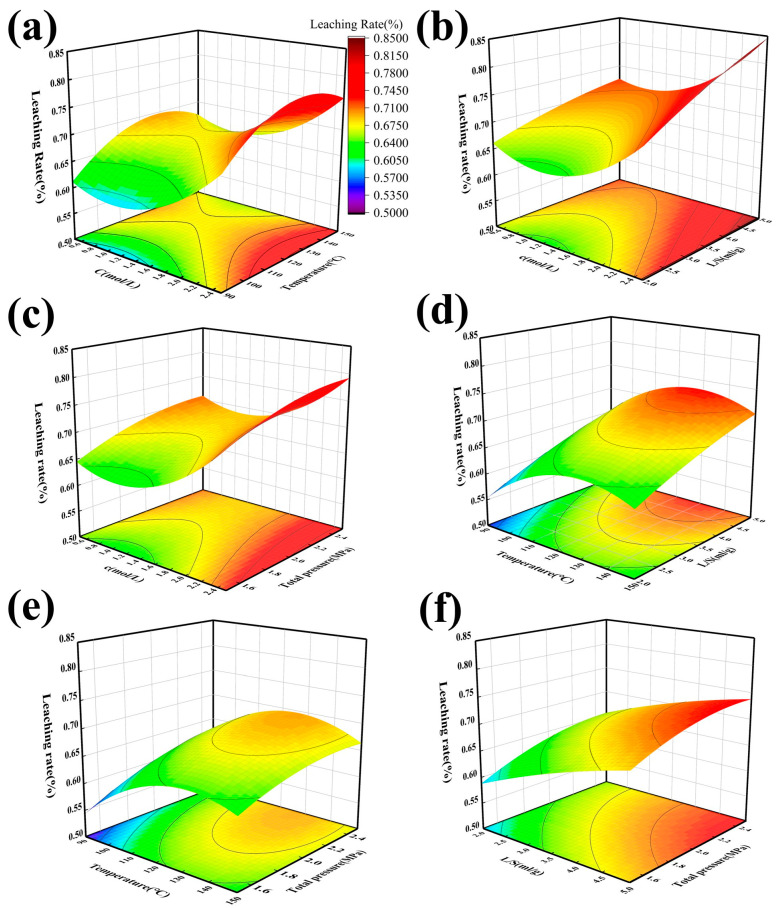
Response surface plots under an acid leaching system; (**a**) effect of the nitric acid concentration and temperature on the V leaching rate; (**b**) effect of nitric acid concentration and liquid-to-solid ratio on the V leaching rate; (**c**) effect of the nitric acid concentration and total pressure on the V leaching rate; (**d**) effect of temperature and liquid-to-solid ratio on the V leaching rate; (**e**) effect of temperature and total pressure on the V leaching rate; and (**f**) effect of liquid-to-solid ratio and total pressure on the V leaching rate.

As illustrated in the 3D response surfaces and contour plots from Figure 11a–f, significant interactions exist between the key variables affecting vanadium leaching efficiency. Figure 11a shows that the interaction between nitric acid concentration and temperature reveals that as both increase, the leaching efficiency improves notably. When the nitric acid concentration surpasses 1.5 mol/L and the temperature reaches approximately 150 °C, the leaching efficiency reaches its peak, highlighting the pivotal role of the oxidative capability of nitric acid in facilitating vanadium dissociation within this range. Figure 11b shows that the interaction between nitric acid concentration and liquid-to-solid ratio enhances the contact area between the acid and the minerals, thereby facilitating vanadium dissolution. Similarly, Figure 11c demonstrates that the interaction between nitric acid concentration and total pressure reveals that elevated oxygen pressure further boosts the oxidative capacity of nitric acid, significantly improving vanadium extraction. Figure 11d–f illustrate the combined effect of temperature, liquid-to-solid ratio, and total pressure, confirming that higher temperatures and optimized oxygen pressure conditions substantially increase leaching efficiency, thereby emphasizing the synergistic role of these factors in vanadium extraction.

Based on the response surface optimization results, and considering the potential for equipment corrosion due to excessively high acidity, the nitric acid concentration is maintained below 1.5 mol/L, with the total pressure kept under 2 MPa. The optimal leaching conditions were chosen as follows: a nitric acid concentration of 1.5 mol/L, a temperature of 127.43 °C, a total pressure of 2 MPa, and a liquid-to-solid ratio of 5 mL/g. Under these conditions, the theoretical leaching rate was 73.1%. After three repeated experiments, the actual leaching efficiency was 78.7%.

#### 3.3.8. Cyclic Leaching Performance

In practical applications, the nitrogen oxide (NO_x_) generated during nitric acid leaching can react with oxygen and water to regenerate nitric acid, providing a feasible pathway for nitrogen recycling and enabling the reuse of nitric acid within the leaching system. The negative Gibbs free energy values of Equations (10)–(12) in Table 4 and Figure 9 confirm the thermodynamic feasibility of this process. To assess the industrial applicability of nitric acid oxygen pressure leaching for stone coal, a cyclic leaching procedure was designed. After each cycle, the free H^+^ concentration in the leachate was measured by acid–base titration, and sulfuric acid was subsequently added to maintain the target H^+^ concentration at 1.5 mol/L. In each cycle, the H^+^ concentration decreased from 1.5 mol/L to approximately 0.8 mol/L after leaching. Simultaneously, ion chromatography combined with solid-phase extraction was employed to monitor the nitrate concentration in the leachate, ensuring effective regeneration and reuse of nitric acid. Subsequently, the acid-adjusted leachate was reused directly in subsequent leaching cycles with fresh ore.

**Figure 12:**
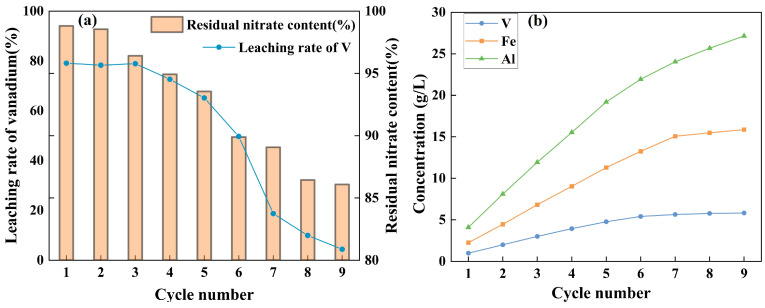
Cyclic leaching performance during nitric acid oxygen pressure leaching of stone coal. (**a**) Variation in vanadium leaching efficiency and residual nitrate content in the leachate over successive cycles. (**b**) Accumulation of metal ions (V, Fe, and Al) in the leachate during repeated leaching.

As shown in Figure 12, the vanadium leaching efficiencies in the first five cycles were 79.1%, 78.3%, 78.9%, 72.6%, and 65.1%, respectively, indicating overall stability. However, starting from the sixth cycle, the leaching efficiency declined markedly, reaching only 4.4% by the ninth cycle. This decline likely results from the gradual accumulation of metal ions, which form precipitates with acid anions, reducing effective acidity, as well as the depletion of easily soluble components and buildup of insoluble fractions in the ore. Despite this decline, the robust performance observed in the early cycles underscores the potential of this method for industrial application, presenting a sustainable and economically viable approach for processing stone coal.

### 3.4. Preliminary Estimation of Reagent Costs in the Nitrogen Recycling System

To preliminarily assess reagent costs under the proposed nitrogen recycling system, oxygen consumption was estimated based on its stoichiometric reaction with the main reductive components in stone coal, including vanadium (assumed as V^3+^) and FeS_2_. Additionally, reagent dosages of nitric acid and sulfuric acid were calculated assuming five successive leaching cycles, consistent with the experimental design described in Section 3.3.8. Therefore, processing 1000 kg of stone coal would require approximately 139.4 kg of nitric acid, 22.9 kg of oxygen, and 274.4 kg of sulfuric acid to compensate for acid loss during recycling. The corresponding reagent costs are approximately CNY 270 for nitric acid, CNY 120 for oxygen, and CNY 247 for sulfuric acid (Table 8). This cost-efficient profile, combined with the elimination of high-temperature roasting and the potential reuse of leaching solution, underscores the economic advantage and sustainability of the proposed process.

## 4. Conclusions

This study introduces a novel vanadium extraction method for stone coal that leverages nitrogen cycling, integrating nitric acid oxygen pressure leaching with nitric acid recycling. This approach boosts vanadium leaching efficiency while cutting nitric acid consumption. The main conclusions are as follows:

1. Single-factor experiments revealed that nitric acid concentration, temperature, liquid-to-solid ratio, and total oxygen pressure markedly influence vanadium extraction efficiency. Through response surface optimization, the optimal conditions were identified as nitric acid concentration of 1.5 mol/L, temperature of 127.43 °C, liquid-to-solid ratio of 5 mL/g, and total pressure of 2 MPa, achieving a vanadium leaching efficiency of 73.1%.
2. The cyclic leaching experiments demonstrated effective nitrate recycling by converting nitrogen oxide (NO_x_) generated during leaching back into nitric acid. This approach not only enhances vanadium leaching efficiency but also significantly reduces nitric acid consumption and wastewater discharge. Cyclic tests conducted under the optimized conditions identified by response surface methodology showed that the system maintains robust reactivity over multiple cycles, indicating strong process stability and sustainability. These findings further confirm the feasibility and industrial applicability of this method.
3. Mineralogical analyses using SEM-EDS, BET, FTIR, and XRD confirmed that nitric acid oxygen pressure leaching effectively disrupts the muscovite structure. Its significant effects on mineral surface morphology, specific surface area, and crystal structure were clearly demonstrated. The oxidative action of nitric acid disrupts the muscovite lattice, forming a porous structure and enlarging the specific surface area, which in turn facilitates the efficient leaching of vanadium from stone coal.

This study provides a sustainable, environmentally responsible method for extracting vanadium from low-grade stone coal, significantly reducing energy consumption and mitigating wastewater challenges, while delivering notable environmental and economic benefits.

## Figures and Tables

**Table 1:** Chemical composition analysis of stone coal.

Composition	SiO_2_	Al_2_O_3_	V_2_O_5_	K_2_O	Na_2_O	MgO	TFe
Content/%	66.34	2.87	0.82	0.37	0.86	0.55	0.93
Composition	CaO	BaO	ZnO	Water	C	S	Loss
Content/%	0.70	0.28	0.05	2.61	19.3	4.32	14.49

**Table 2:** Comparison of leaching under different conditions.

No.	Leaching Condition	Acid Type	Gas Atmosphere, Total Pressure	Remarks
1	Nitric acid oxygen pressure leaching	2 mol/L nitric acid	O_2_, 2 MPa	Reference condition
2	Non-assisted nitric acid leaching	2 mol/L nitric acid	residual air	No gas injection
3	Nitric acid nitrogen pressure leaching	2 mol/L nitric acid	N_2_, 2 MPa	Evaluates effect of gas type
4	Sulfuric acid oxygen pressure leaching	1 mol/L sulfuric acid	O_2_, 2 MPa	Evaluates effect of acid type; constant initial H^+^ concentration

**Table 3:** Pore parameters of raw ore and leaching residues.

Samples	Specific Surface BET (m^2^/g)	Total Pore Volume (cm^3^/g)	BJH Average Aperture (nm)
Raw ore	0.951	0.00873	3.092
Leaching residue with H_2_SO_4_	4.274	0.08412	3.925
Leaching residue with HNO_3_	32.297	0.05804	3.912

**Table 4:** Equations representing various reactions and the standard Gibbs free energy variations as a function of temperature.

Chemical Reaction	Gibbs Free Energy Change Relationship to Temperature (kJ/mol)	Equation
3V_2_O_3(s)_ + 2NO_3_^−^_(aq)_ + 14H^+^_(aq)_ = 6VO^2+^_(aq)_ + 2NO_(g)_ + 7H_2_O_(l)_	ΔG = −735.12 + 0.4978T	(3)
2V_2_O_3(s)_ + O_2(g)_ + 8H^+^_(aq)_ = 4VO^2+^_(aq)_ + 4H_2_O_(l)_	ΔG = −652.12 + 0.6571T	(4)
2H^+^_(aq)_ + FeS_2(s)_ = H_2_S_(g)_ + Fe^2+^_(aq)_ + S_(s)_	ΔG = 60.213 − 0.084T	(5)
H_2_S_(g)_ + 2O_2(g)_ = ${\mathrm{SO}}_{4}^{2-}$_(aq)_ + 2H^+^_(aq)_	ΔG = −889.10 + 0.5972T	(6)
3H_2_S_(g)_ + ${8\mathrm{NO}}_{3}^{-}$_(aq)_ + 2H^+^_(aq)_ = 8NO_(g)_ + 4H_2_O_(l)_ + 3${\mathrm{SO}}_{4}^{2-}$_(aq)_	ΔG = −1695.11 − 0.1598T	(7)
FeS_2(s)_ + 5${\mathrm{NO}}_{3}^{-}$_(aq)_ + 4H^+^_(aq)_ = Fe^3+^_(aq)_ + 5NO_(g)_ + 2${\mathrm{SO}}_{4}^{2-}$_(aq)_ + 2H_2_O_(l)_	ΔG = −918.630 − 0.222T	(8)
4FeS_2(s)_ + 15O_2(g)_ +2H_2_O_(l)_ = 4Fe^3+^_(aq)_ + 8 ${\mathrm{SO}}_{4}^{2-}$_(aq)_ + 4H^+^_(aq)_	ΔG = −6115.45 + 4.029T	(9)
4NO_(g)_ + 3O_2(g)_ + 2H_2_O_(l)_ = 4H^+^_(aq)_ + 4 ${\mathrm{NO}}_{3}^{-}$_(aq)_	ΔG = −486.09 + 0.9757T	(10)
2NO_(g)_ + O_2(g)_ = 2NO_2(g)_	ΔG = −114.40 + 0.1463T	(11)
4NO_2(g)_ + O_2(g)_ + 2H_2_O_(l)_ = 4H^+^_(aq)_ + 4 ${\mathrm{NO}}_{3}^{-}$_(aq)_	ΔG = −257.29 + 0.6831T	(12)
BaCO_3(s)_ + 2H^+^_(aq)_ = CO_2(g)_ + Ba^2+^_(aq)_ + H_2_O_(l)_	ΔG = −0.1353 − 0.1800T	(13)
KAl_2_Si_3_AlO_10_(OH)_2(s)_ + 10H^+^_(aq)_ = K^+^_(aq)_ + 3Al^3+^_(aq)_ + 3H_2_SiO_3(s)_ + 3H_2_O_(l)_	ΔG = −307.57 + 0.5687T	(14)

**Table 5:** Range of independent factors and their levels in the leaching system.

Independent Factors	Range and Levels				
	−2	−1	0	+1	+2
A: HNO_3_ concentration (mol/L)	/	0.5	1.5	2.5	3.5
B: Leaching temperature (°C)	70	90	120	150	170
C: Liquid–Solid (mL/g)	/	2	3.5	5	6.5
D: Total pressure (MPa)	/	1.5	2	2.5	3

**Table 6:** Results of Central Composite Design response surface experiments.

Std	Run	A: c	B: T	C: L/S	D: Total Pressure	Y: Leaching Rate
1	25	0.5	90	2.0	1.5	0.5086
2	10	2.5	90	2.0	1.5	0.5845
3	6	0.5	150	2.0	1.5	0.6105
4	20	2.5	150	2.0	1.5	0.6649
5	19	0.5	90	5.0	1.5	0.5905
6	4	2.5	90	5.0	1.5	0.7210
7	11	0.5	150	5.0	1.5	0.6658
8	8	2.5	150	5.0	1.5	0.7913
9	7	0.5	90	2.0	2.5	0.6104
10	15	2.5	90	2.0	2.5	0.6613
11	27	0.5	150	2.0	2.5	0.6445
12	18	2.5	150	2.0	2.5	0.7154
13	1	0.5	90	5.0	2.5	0.6658
14	3	2.5	90	5.0	2.5	0.7856
15	24	0.5	150	5.0	2.5	0.6991
16	14	2.5	150	5.0	2.5	0.8156
17	26	3.1	120	3.5	2.0	0.9084
18	13	1.5	70	3.5	2.0	0.5074
19	21	1.5	170	3.5	2.0	0.5921
20	2	1.5	120	6.5	2.0	0.7537
21	17	1.5	120	3.5	3.0	0.6849
22	12	1.5	120	3.5	2.0	0.6856
23	16	1.5	120	3.5	2.0	0.6901
24	5	1.5	120	3.5	2.0	0.6820
25	22	1.5	120	3.5	2.0	0.6902
26	9	1.5	120	3.5	2.0	0.6945
27	23	1.5	120	3.5	2	0.6894

**Table 7:** Results of the CCD experiments on the vanadium leaching rate.

Source	Sum of Squares	df	Mean Square	F-Value	*p*-Value	
Model	0.1988	14	0.0142	396.59	<0.0001	Significant
A-C	0.0368	1	0.0368	1028.74	<0.0001	
B-T	0.0179	1	0.0179	498.86	<0.0001	
C-L/S	0.0351	1	0.0351	980.90	<0.0001	
D-P	0.0139	1	0.0139	387.23	<0.0001	
AB	0.000006	1	0.000006	0.1676	0.6895	
AC	0.0036	1	0.0036	100.69	<0.0001	
AD	0.0000	1	0.0000	1.39	0.2616	
BC	0.0002	1	0.0002	6.62	0.0244	
BD	0.0019	1	0.0019	54.30	<0.0001	
CD	0.0003	1	0.0003	7.51	0.0179	
A^2^	0.0252	1	0.0252	704.04	<0.0001	
B^2^	0.0350	1	0.0350	976.03	<0.0001	
C^2^	0.0007	1	0.0007	20.06	0.0008	
D^2^	0.0035	1	0.0035	97.26	<0.0001	
Residual	0.0004	12	0.000033			
Lack-of-Fit	0.0003	7	0.000043	2.46	0.1697	Not significant
Pure Error	0.0001	5	0.00002			
Cor Total	0.1993	26				

**Table 8:** Estimated reagent cost under the nitrogen recycling system (per 1000 kg of stone coal).

Reagent	Amount of Substance (mol)	Mass (kg)	Estimated Total Cost (CNY)
Oxygen	715	22.9	120
Nitric acid	1500	139.4	270
Sulfuric acid	2800	274.4	247

## Data Availability

The original contributions presented in this study are included in the article. Further inquiries can be directed to the corresponding author(s).
